# Supplementary material for: Discovery of a Role for Rab3b in Habituation and Cocaine Induced Locomotor Activation in Mice Using Heterogeneous Functional Genomic Analysis
Source: Front Neurosci. 2020 Jul 9;14:721. doi: 10.3389/fnins.2020.00721 (PMC7364128; doi:10.3389/fnins.2020.00721)
Supplement: Supplementary file 2 [file Presentation_1.pdf]

Supplemental Methods:

Mutant mice, B6;129-*Rab3b*^{tm1Sud} *Rab3dt*^{m1Rja} *Rab3c* ^{tm1Sud}/J carrying targeted mutations in *Rab3b*, *Rab3c* and *Rab3d* genes were acquired from the Jackson Laboratory Repository (JR#006375). The production of these mice is described by Schulter et al, 2004 (PMID:15269275). Chimeric mice were mated to C57BL/6 mice and the compound mutants created by intercrossing individual lines. The strain was backcrossed 2 or 3 times to C57BL/6 before being deposited in the repository. The *Rab3b*^{tm1Sud} allele was isolated from the other alleles by 3 to 4 additional backcrosses to C57BL/6J to produce B6.129-*Rab3b*^{tm1Sud} these were compared to C57BL/6J mice from the colony.

Supplemental Results:

Assessment of Cocaine Response in *Rab3b* null mice

Response to repeated exposure to cocaine in single mutant

A repeated measures analysis of variance approach was used to assess locomotor response following repeated exposure to cocaine (repeated factor being Day) No significant effect of day x genotype x treatment x sex observed $F_{(3,52)}$=0.116, p <0.1228 nor was an effect of day x genotype x treatment observed $F_{(3,56)}$ = 0.003949, p<0.9738.

Acute response to cocaine

Analysis of acute response to cocaine (Day3 – Day 2) showed no effect of Genotype on the acute response to cocaine ($F_{(1,29)}$ = 1.6026, p < 0.2160). To control for habituation, Cocaine was also administered to a sub group on day 1 and compared to the Saline mice from Day 1. There was no effect of treatment x genotype ($F_{(1,37)}$= 0.3257, p < 0.9827) but a strong effect of treatment alone ($F_{(1,39)}$= 139.3721, p < 0.0001)

Sensitized response to cocaine

In addition to assessing the effects of repeated exposure to cocaine in this panel on mice, we also assessed the development of cocaine sensitization. Analysis of cocaine sensitization calculated as Day 9COCA – Day 3COCA locomotion revealed only an effect of treatment ($F_{(1,61)}$=145.2208 p<0.0001)

Supplemental Figure 1. Main and interaction effect plots. A. Effect plot for the peak marker on Chr 4 (rs13477919 @ 104.96 Mb) indicates a significant effect of genotype (p < 0.0003). Furthermore at this locus the D2-derived allele is the increaser allele. B. Effect plot for the peak marker on Chr 15 (rs13482528 @ 38.82 Mb) indicates a significant effect of genotype (p < 0.0001). C. Interaction plot for the significant Chr 4 (rs13477881 @ 103.31 Mb) x Chr 5 (rs3723202 @ 91. 67 Mb) (p < 0.0001). There is a significant difference in the phenotypic values when the genotypes at rs3723202 and rs13482528 are BB, BB and BB, DD, respectively.

Supplemental Figure 2. Cocaine-induced locomotor activation in Rab3b mutants and Rab3b +/+ (C57BL/6J) mice. There were no differences between the Rab3b -/- and the Rab3b +/+ mice in response to cocaine sensitization.

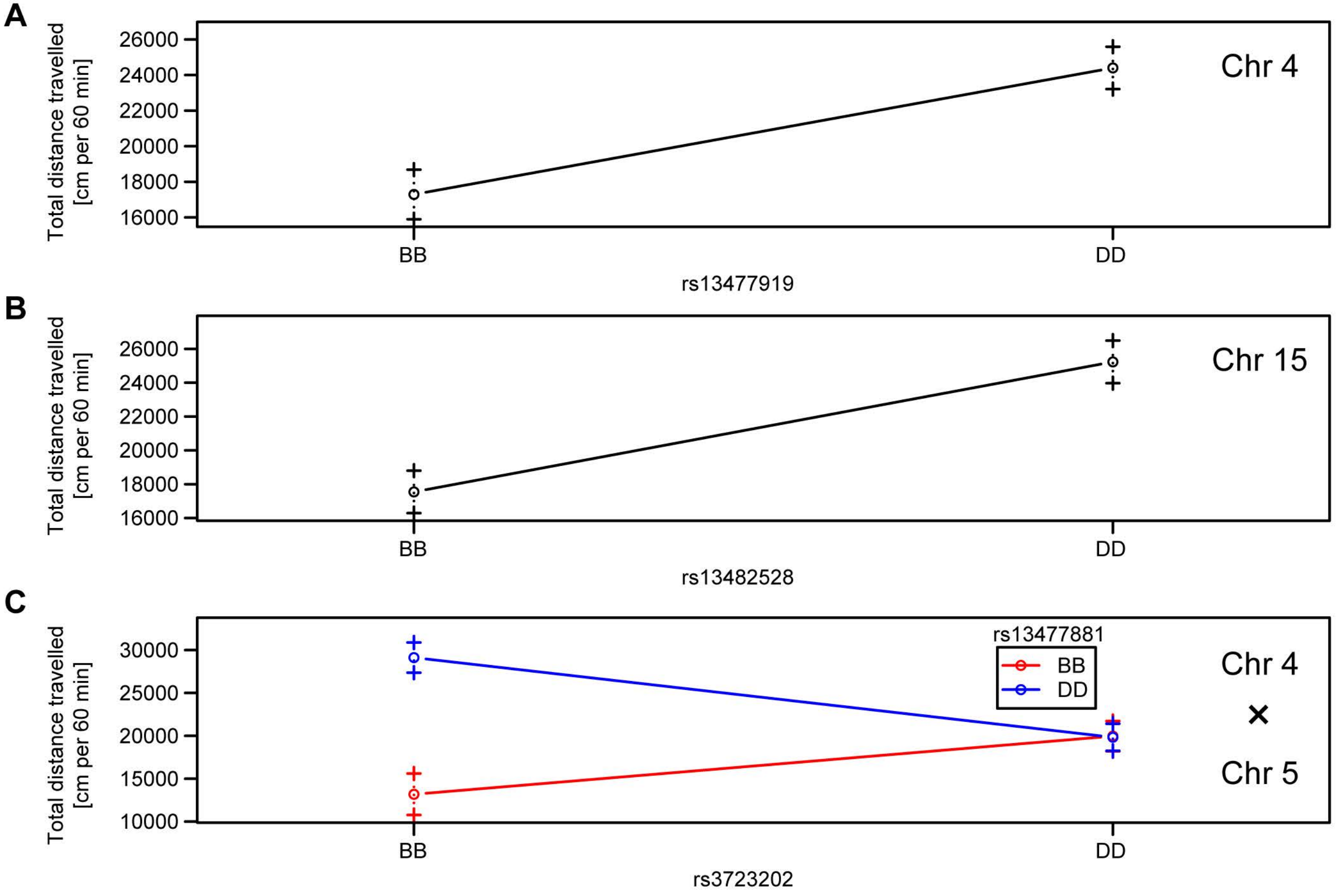
A
Total distance travelled [cm per 60 min]
26000
24000
22000
20000
18000
16000
Chr 4
BB
DD
rs13477919
B
Total distance travelled [cm per 60 min]
26000
24000
22000
20000
18000
16000
Chr 15
BB
DD
rs13482528
C
Total distance travelled [cm per 60 min]
30000
25000
20000
15000
10000
rs13477881
BB
DD
Chr 4
×
Chr 5
BB
DD
rs3723202

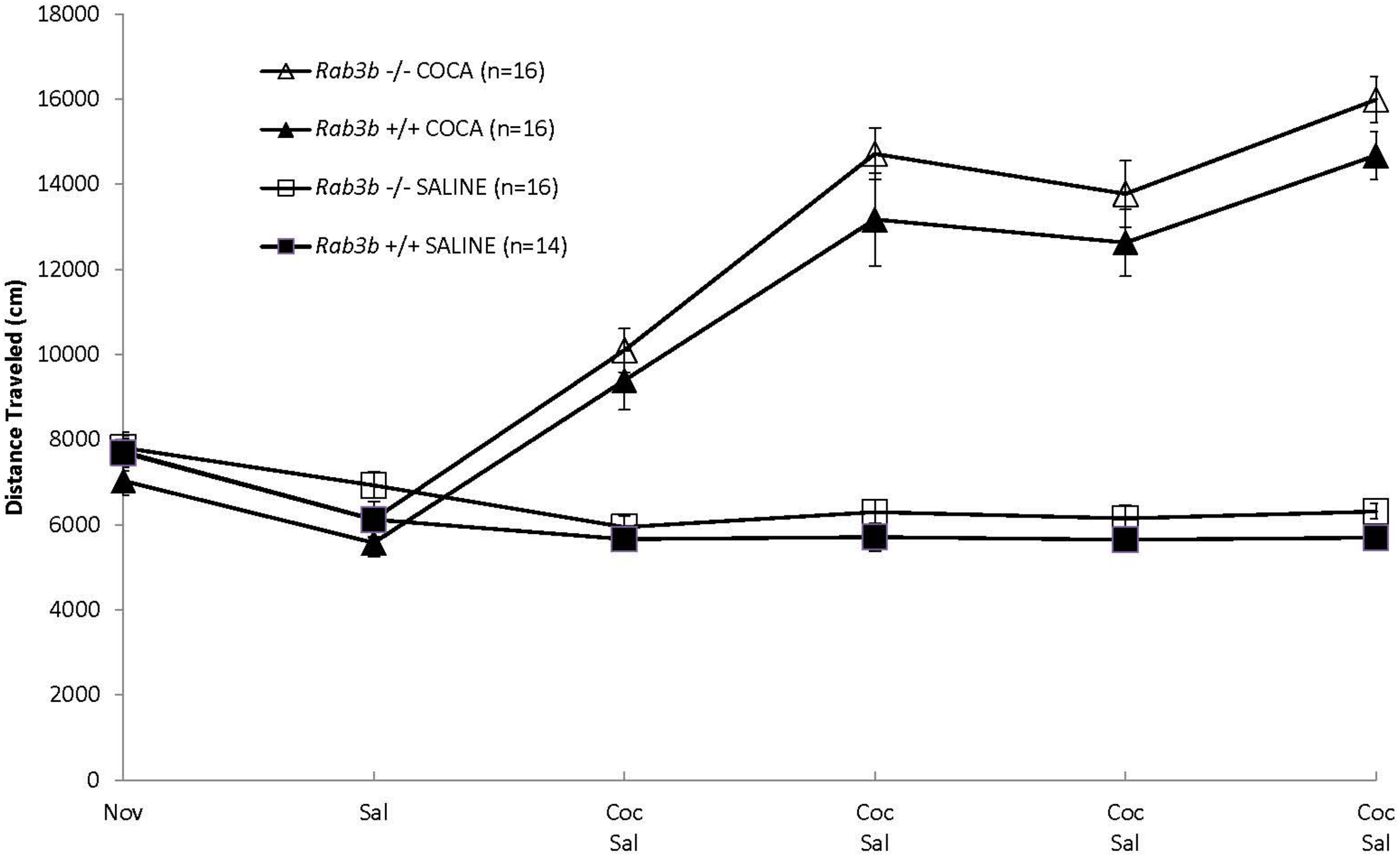

Rab3b -/- COCA (n=16)
Rab3b +/+ COCA (n=16)
Rab3b -/- SALINE (n=16)
Rab3b +/+ SALINE (n=14)
Distance Traveled (cm)
18000
16000
14000
12000
10000
8000
6000
4000
2000
0
Nov
Sal
Coc Sal
Coc Sal
Coc Sal
Coc Sal
